# Plasma levels of the active form of suPAR are associated with COVID-19 severity

**DOI:** 10.1186/s13054-020-03336-0

**Published:** 2020-12-29

**Authors:** Mingxiang Huang, Linlin Li, Jianshan Shen, Yao Wang, Rui Wang, Cai Yuan, Mingdong Huang, Longguang Jiang

**Affiliations:** 1 Department of Clinical Laboratory, Fuzhou Pulmonary Hospital of Fujian, Fuzhou, 350008 Fujian China; 2 College of Chemistry, Fuzhou University, Fuzhou, 350116 Fujian China; 3 College of Biological Science and Engineering, Fuzhou University, Fuzhou, 350116 Fujian China; 4 Fujian Key Laboratory of Marine Enzyme Engineering, Fuzhou University, Fuzhou, Fujian China

We read with great interest the recent study by Rovina et al., who found that the elevation of soluble urokinase plasminogen activator receptor (suPAR) plasma levels in coronavirus disease 2019 (COVID-19) patients, and suggested the suPAR can be an early predictor of severe respiratory failure [1]. There are three different suPAR forms (suPAR DI-III, suPAR DI, and suPAR DII-III) in circulation, in which suPAR DI-III is defined as the active form of suPAR by its capability of binding to uPA [2]. In addition, the uPA/uPAR system as a therapeutic target has been proposed to reduce mortality of COVID-19 [3]; therefore, further evaluation of the active form of suPAR plasma levels in different symptom types of COVID-19 patients and asymptomatic carriers could still provide important indications for required early admission and treatment.

In our study, we found that active suPAR levels in all COVID-19 patients were significantly higher than in healthy controls (5.51 ± 2.53 ng/mL vs 1.97 ± 0.78 ng/mL, *p* < 0.0001) using a ELISA assay modified from our previously reported method [4] where the active suPAR was captured by a uPAR ligand and measured using a polyclonal anti-uPAR antibody. Strikingly, the active suPAR levels in asymptomatic carriers (8.08 ± 4.81 ng/mL) are not only significantly higher than those in healthy controls (*p* < 0.0001) but also slightly higher than those in COVID-19 patients (*p* = 0.0278) (Table 1, Fig. 1a). Even though more data needs to be collected and the background of these patients are not clear, this is a significant research direction to pursue. If asymptomatic carriers could be identified and quarantined in an early stage, it will prevent them from increasing the disease transmission to an uncontrollable manner.

**Table 1:** The active suPAR levels in plasma are associated correlated with high-sensitivity C-reaction protein (hs-CRP), neutrophil/leukocyte ratio, and lymphocyte counts

Patient type	Number of samples	Active suPAR (ng/mL)	hs-CRP (mg/L)	Neutrophil/leukocyte ratio (%)	Lymphocyte (×10^9^/L)
Moderate	57	4.57 ± 2.35	3.01 ± 3.67	59.98 ± 9.34	1.62 ± 0.60
Severe	30	5.97 ± 2.31	38.79 ± 57.81	64.60 ± 11.23	0.97 ± 0.35
Critical	21	6.68 ± 2.54	135.39 ± 93.88	87.49 ± 9.53	0.66 ± 0.37
Asymptomatic	9	8.08 ± 4.81	1.29 ± 1.14	65.78 ± 9.30	1.57 ± 0.49

**Fig. 1:**
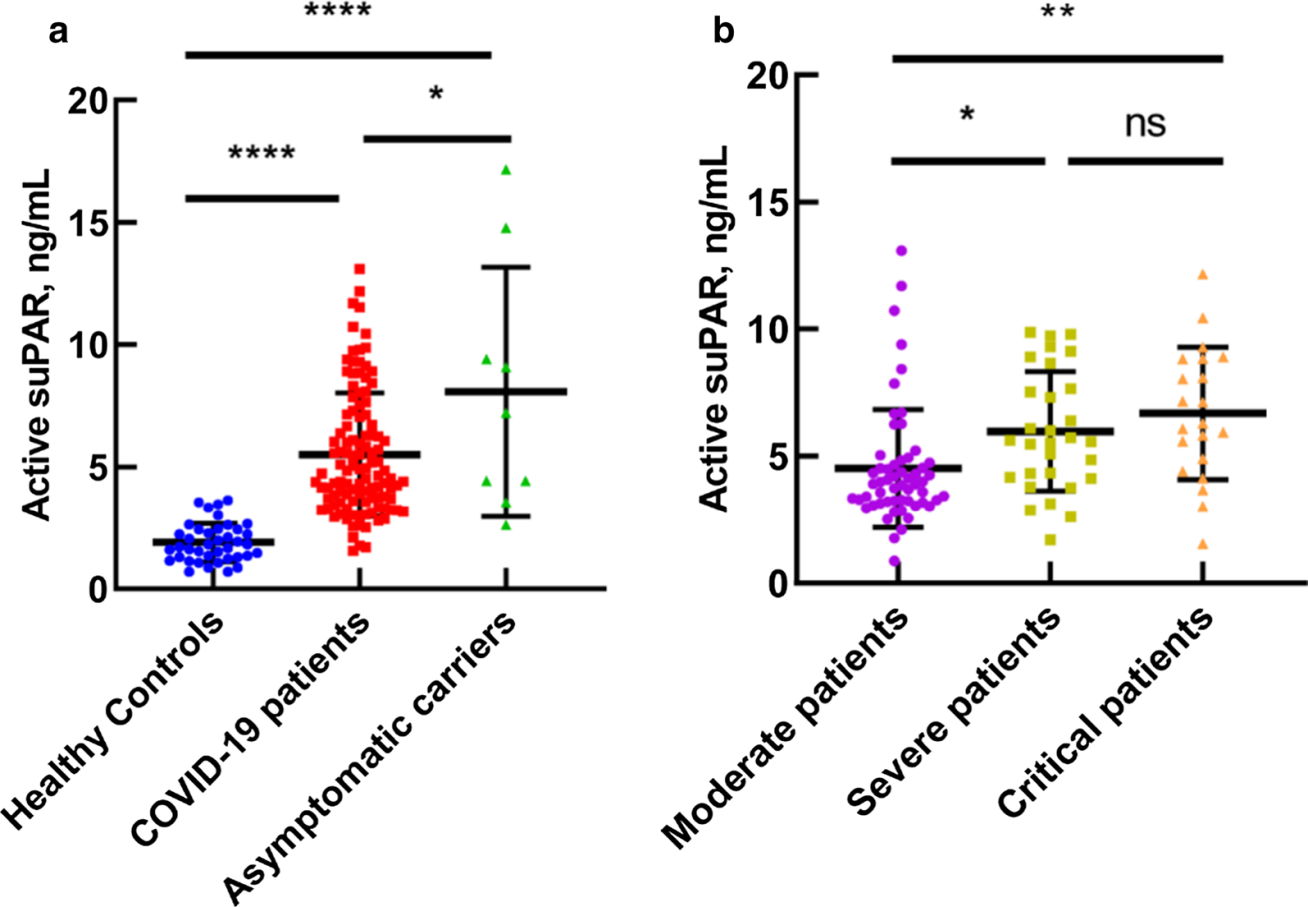
Level of plasma active suPAR in different classifications. **a** Active suPAR level in COVID-19 patients and asymptomatic carriers, compared with active suPAR level in healthy controls. **b** Level of active suPAR between three different COVID-19 classifications including moderate, severe, and critical

Patients involved three types of symptoms: moderate, severe, and critical in our study (Table 1). Our data show that active suPAR levels increase as the disease worsens (Fig. 1b). Moreover, correlation analyses demonstrated that active suPAR levels are positively correlated with high-sensitivity C-reaction protein (hs-CRP), neutrophil/leukocyte ratio, and lymphocyte counts (Table 1).

Therefore, taken together with the results from Rovina et al., these results demonstrated that the active suPAR as a COVID-19 prognostic biomarker may assist in the early triage of SARS-CoV-2-infected persons to prevent virus transmission. Further studies are needed to see whether the elevation of suPAR plasma levels in COVID-19 patients is due to the enhanced over-expression of uPAR or due to their increased shedding from the cell surface.

## Data Availability

The dataset supporting the conclusion of this article is available from the corresponding author upon reasonable request.

## Abbreviations

COVID-19: Coronavirus disease 2019
ELISA: Enzyme-linked immunosorbent assay
hs-CRP: High-sensitivity C-reaction protein
SARS-CoV-2: Severe acute respiratory syndrome coronavirus 2
suPAR: Soluble urokinase plasminogen activator receptor
uPA: Urokinase plasminogen activator
