# Supplementary material for: Plasma levels of the active form of suPAR are associated with COVID-19 severity
Source: Crit Care. 2020 Dec 29;24:704. doi: 10.1186/s13054-020-03336-0 (PMC7770382; doi:10.1186/s13054-020-03336-0)
Supplement: Related texts

# Reply: Authors’ response

**Authors:** Jesper Eugen-Olsen, Salim Hayek, Jochen Reiser, Evangelos J. Giamarellos-Bourboulis

**Affiliations:** 5 grid.411905.8 0000 0004 0646 8202 Clinical Research Centre, Copenhagen University Hospital Hvidovre, Hvidovre, Denmark; 6 grid.214458.e 0000000086837370 Department of Medicine, University of Michigan, Ann Arbor, MI 48103 USA; 7 grid.240684.c 0000 0001 0705 3621 Department of Medicine, Rush University Medical Center, Chicago, IL 60612 USA; 8 grid.5216.0 0000 0001 2155 0800 4th Department of Internal Medicine, Medical School, National and Kapodistrian University of Athens, 11527 Athens, Greece; 9 grid.411449.d 0000 0004 0622 4662 4th Department of Internal Medicine, ATTIKON University Hospital, 1 Rimini Street, 12462 Athens, Greece

The urokinase plasminogen activator receptor (uPAR) is an immune cell expressed GPI-linked receptor that may be cleaved from the cell surface generating soluble uPAR (suPAR). There are three forms of the suPAR molecule, the full length 3-domain protein (DIDIIDIII), and the cleaved forms (DIIDIII) and DI; only DIDIIDIII can bind uPA with high affinity generating soluble DIDIIDIII/uPA complexes [5]. Both the full length and DIIDIII molecules have been shown to carry independent prognostic value [6].

Huang et al. report that “active suPAR” increases with severity of COVID-19,” but surprisingly find that the level is even higher among some of the 9 included asymptomatic carriers. As part of our “International consortium on inflammation in COVID-19,” we have tested almost 1000 COVID-19 patients for suPAR. Among patients with no or mild symptoms of infection, we find average levels about 4 ng/ml, much lower than those that are hospitalized [7]. To further confirm this finding, we measured suPAR in the plasma of 11 patients with very mild symptoms. All were adults who provided written informed consent with molecular identification of SARS-CoV-2 by routine real-time PCR in the nasopharyngeal secretions sampled by a standard collection swab. Blood sampling was done within 15 min after swab collection; median suPAR was 2.0 ng/ml (range 1.70–5.21 ng/ml).

In the above studies of our group, but also in the vast majority of published studies so far, suPAR is measured by the suPARnostic® assays. These assays are using two monoclonal antibodies (developed into lateral flow quick tests, enzyme immunoassays, and turbidimetric assays on Roche, Abbott, and Siemens platforms) and are the only CE/IVD approved suPAR assays for clinical decision making on the market.

Why this difference between the suPARnostic® assays and the enzyme immunoassay is described by Huang et al.? Likely, because the suPARnostic® assays are developed based on prognostic value using combinations of antibodies on plasma from patients with known disease progression to identify antibodies that bind the suPAR molecules with the highest clinical value. On the contrary, the assay described by Haung et al. may capture only a subgroup of the prognostic relevant suPAR molecules.

In conclusion, our data using the suPARnostic® assays both in a large published cohort of patients [1, 7] and in a recent cohort of mild cases from Greece support that patients with asymptomatic or mild COVID-19 have lower levels of suPAR than severe cases.
